# Psychological mechanisms of revenge and revenge ideations in family homicide: results from qualitative research of forensic assessment reports

**DOI:** 10.1186/s12888-025-07021-w

**Published:** 2025-06-05

**Authors:** L. H. Grobbink, K. M. L. Huijbregts, S Draisma, J. J. L. Derksen, G. J. Westerhof

**Affiliations:** 1 GGZ Oost Brabant mental health care, Postbus 3, 5427 ZG Boekel, the Netherlands; 2 Scelta/GGNet NL, Warnsveld, the Netherlands; 3 Trimbos Institute, Utrecht, the Netherlands; 4 Radboud University Nijmegen, Nijmegen, the Netherlands; 5 University of Twente, Enschede, the Netherlands

**Keywords:** Revenge, Vengeance, Personality, Family homicide, Psychopathology, Qualitative research, Forensic assessment reports

## Abstract

Our aim was to find out which social and psychological factors characterize forensic psychiatric patients who have committed family homicide with revenge as a reason as compared to subjects who committed family homicide with other motives. Qualitative research was carried out on the basis of pre-trial forensic assessment reports of existing cases (*N*=20), divided between Revenge and No-Revenge cases. In case of revenge, violence was almost always a sort of settling of an interpersonal score. Psychotic symptomatology was absent in the Revenge cases, personality problems (particularly borderline and narcissistic traits) were common. Demoralization because of a decline of well-being seems to be an important factor pushing some persons with such vulnerabilities over the edge. Our expectation is that, at least in a certain proportion of (non-psychotic) patients, there will be more brooding on revenge than the psychotherapist suspects.

In 2013, Jeroen D. killed his nine- and seven-year old sons, after which he killed himself. He had prepared his act thoroughly and the probable cause was hearing the court-ruling of a restricted contact arrangement [1]. The media quickly concluded revenge must have played a role in this dreadful killing. However, the extensive media attention devoted to these and similar cases is in sharp contrast to the number of scientific studies on revenge. Due to this lack of research, there are no widely-shared diagnostic and treatment programs for revengeful patients. Better understanding of revenge and the development of revenge ideations and feelings may lead to improvement of treatment plans and policies, and may prevent escalations and acting-out behaviors.

Revenge can be defined as a harmful action in response to feeling offended or wronged that has the function of restoring psychological balance and sense of self [2]. In a previous attempt at conceptualizing revenge, we found that revenge may be a pursuit [2], which develops after a certain strain and is intrapersonal because of its cognitive, introverted nature. Revenge can be directed inward (toward oneself) or outward (toward the other) and can substantially increase over time. Revenge extends beyond anger as becoming outraged will not restore the disturbed psychological balance after an injury. It can be conceptualized as behavior following revenge ideations [2, 3]. Revenge ideations refer to psychological processes, such as fantasies, thoughts and feelings, in which rumination, cognitive distortions (tunnel vision, all-or-nothing thinking and overgeneralization) take place. Cognitive distortions contribute to the threatening image of “the other”, who needs to be avenged or eliminated. This is dependent on the personality of the avenger and the function revenge ideations and ruminations have. Fantasizing about revenge is accompanied by feelings of authority and omnipotence and offers the possibility to identify with violent characters [4, 5]. It is important to note that revenge ideation does not necessarily lead to actual revenge (behavior). Research addressing the interplay between both factors is therefore very important.

Research has shown that certain psychological symptoms can be associated with an increased desire for revenge, such as symptoms of PTSD [6], anxiety, and depression [7, 8]. On the level of personality, revenge ideation could also be part of a narcissistic personality disorder or a borderline personality disorder [3, 9]. Early problems in development (e.g., traumas, hostility, or “being ignored”) seem to be important in such cases. Psychodynamically, revenge could serve the purpose of holding on to the psychological object [10]. It could therefore play a role in attempting to manage a conflict of distance and proximity when a real or imaginary threat, abandonment, or rejection by a significant other is present [11]. Marital problems and/or doubts about fatherhood in combination with borderline personality problems and/or a high degree of narcissism, are factors that specifically play a role in revenge in partner and/or child murders [12]. In the context of marital problems, the offender can also see children as the source of hostility and anger, especially when there are doubts about fatherhood. Threats from a spouse to leave a person (usually a male, [12]) or an obstruction to have contact with children, can cause anger, jealousy and rage directed towards the children. There is also often a lack of individuation on the part of the offender, and an inability to accept a divorce [13]. Also, studies have identified a strong relation between trait-level anger and revenge [14].

It is crucial to understand the psychological processes underlying intense revenge ideation and its escalation into homicide. This understanding enables clinicians to identify warning signs (red flags) in conjunction with contextual triggers.

In this study, we investigated which themes played a role in the case files of individuals who had killed their partner and/or child (family homicide). Since we aimed to understand which themes were specific to the group that had revenge as a motive, we also studied case files where revenge was not a motive.

Records are available at the Netherlands Institute of Forensics Psychiatry and Psychology (NIFP) which allow for in-depth exploration of the phenomenon using qualitative research techniques. To our knowledge this is the first study in which this methodology was used.

This article focuses on the question: which themes emerge in the case files of individuals who have killed their partner and/or child out of revenge compared to those who committed this act for other reasons?

This research question can be further specified through the following subquestions:

1. Which mental health disorders were identified in the suspects?
2. What information is available regarding their personality development?
3. What is known about their personality at the time of the act?
4. Which other factors may have played a role (e.g., substance abuse or contextual factors)?

## Methods

### Design

Qualitative research was carried out on the basis of pre-trial forensic assessment reports of existing cases. Data were organized theme wise in data capture sheets to enable systematic comparisons between two group of suspects: a group with and a group without revenge motives. Although the trial was yet to come, all suspects in this investigation have confessed to the (attempted) murder. The cases were obtained from archives of the Netherlands Institute of Forensics Psychiatry and Psychology (NIFP).

### Setting

In many countries, persons prosecuted for a serious offense, are required to participate in a pre-trial forensic assessment at the request of the judiciary. In a number of countries, these assessments are mainly directed at the establishment of the competence to stand trial (adjudicative competence) and at the restoration of the abilities associated with the competency to stand trial (competence restoration) [15, 16]. For an extensive overview of the impact of legal traditions see Beis et al. [17].

In the Netherlands, where the data for this study was collected, pre-trial forensic assessments focus solely on (possible grounds for excluding) criminal responsibility, risk assessment, and providing advice on treatment and/or probation to minimize recidivism. Forensic pre-trail assessments are conducted by an independent forensic expert (forensic psychiatrist or psychologist), who writes a pre-trial forensic mental health report (further referred to as “pre-trial forensic report”) in which four forensic questions are addressed: (1) whether and to what extent a mental disorder or deficient mental development is present; (2) whether and to what extent a mental disorder or deficient mental development resulted in an advice on the defendant’s diminished criminal responsibility; (3) what the probability is of reoffending (risk assessment); and (4) which judicial and therapeutic measures are needed to reduce the risk of reoffending (risk management). Although each forensic question addresses a distinct forensic aspect, these questions are interconnected and logically sequenced [18, 19].

The cases were obtained from archives of the Netherlands Institute of Forensic Psychiatry and Psychology (NIFP). Forensic assessments can consist of one report (by a psychiatrist or a psychologist), two reports (psychiatrist and psychologist) or three reports (psychiatrist, psychologist, and social worker). These experts (psychiatrists, psychologists, and social workers) can take considerable time for diagnostics, given that the issues are often very complex and the stakes for both the suspects and society are high. To support this, experts undergo a special training program and regularly attend refresher courses and reference meetings to stay updated on the latest insights.

The authors of this article only read the reports, were not involved in writing them, and did not interview the participants. The reports, including the DSM-IV(-TR) classifications made in the reports, were made by classified professionals (psychiatrists and psychologists). In the Netherlands psychologists with post-academic training are well trained to use the DSM criteria, and are qualified to classify according to this system. The forensic assessments found contained psychological reports, psychiatric reports, and integrated psychological and psychiatric forensic reports.

The details regarding the frequency and duration of meetings with the offenders were not clearly specified for each case and it is difficult to provide the exact hours spend, so the following representations of hours that were spend are estimates based on the research times that could be found in the reports. In three of the Non-Revenge cases, only “multiple meetings” was noted. For the remaining seven Non-Revenge cases, the number of meetings varied from two to eight, excluding additional conversations with a social worker (no specific durations were provided). The shortest total research time was 4 hours, while the longest was 18 hours. In the Revenge cases, four cases also only mentioned “multiple meetings,” while the remaining six varied between two and nine meetings, also excluding additional conversations with a social worker . The total research durations ranged from a minimum of 4 hours to a maximum of 19 hours. Research methods included for both cases multiple questionnaires and research methods (frequently: personality questionnaires, neuropsychological research, intelligence research, complaint lists, coping, projective techniques, questionnaires on malingering, risk assessment. On indication: questionnaires on dissociation, social and emotional intelligence, autism, ADHD, and alexithymia questionnaires, biographical questionnaires, multimodal anamnesis).

### Case selection

Our qualitative investigation was carried out on the case histories of 20 persons who committed filicide (seven cases No Revenge, four cases Revenge), uxoricide (two cases No Revenge, five cases Revenge) and familicide (one case No Revenge, one case Revenge). The willingness of the suspect to participate in the psychological investigation and the confession of their act were conditions for inclusion. Between 2014 and 2016, pre-trial forensic assessments of 30 persons were found concerning familicide, uxoricide, and filicide. The forensic assessments were conducted between 2006 and 2016.

The cases were classified by the first author (LG) as either ‘Revenge’ or ‘No Revenge’. In order to determine whether the behavior that led to the offense in the case in question was revenge, it was compared with the following definition: “Behavior following revenge ideations (revenge fantasies, revenge thoughts, feelings of revenge), in which rumination, cognitive distortions (tunnel vision, all-or-nothing thinking and overgeneralization) took place as well as fantasies that arose after being offended or wronged (in the perception of the revenge taker), and in which revenge has the function of restoring the psychological imbalance and the sense of self ” [2]. This is based on our underlying theories [2, 3] of revengefulness as both a pursuit and an intrapersonal phenomenon that has the potential to be destructive. While not inherently pathological, it depends on the personality of the avenger and the functions of his revenge ideations and ruminations, which are, among other things, often linked to early problems in development. See also Boon and Yoshimura [20], who wrote that revenge ought to be considered neither good or bad in and of itself. They argue that a full understanding of revenge requires investigation from all angles, both its destructive and constructive outcomes and consequences.

The suspects in the No Revenge group had also killed or attempted to kill their partner and/or their child, because of an escalation of an argument, assisted suicide, or in case of psychosis or a culturally-colored experience, such as the influence of a Djinn, the Devil or witchcraft.

Case-files of ten subjects could be assigned to the category ‘revenge as motive’ without any doubt, whereas another ten arguably concerned other motives than revenge. The case files of the remaining ten subjects could not be assigned to either revenge or no revenge motives and were disregarded.

In summary, ten cases with revenge as motive versus ten cases with another motive were used in the analysis. So the eventual sample concerned 20 unique persons, who were examined several times by forensic psychologists and psychiatrists.

### Suspect characteristics

Case histories of 20 persons who committed filicide, uxoricide or familicide were included in the analysis. Demographics of the suspects are shown in Table 1.

**Table 1:** Suspect characteristics

	**Revenge (*N*=10)**	**No Revenge (*N*=10)**
Age, x̄ and SD	40,5 (12,7)	40,1 (9,4)
Sex		
Female	2	7
Male	8	3
Ancestry/geographic background		
European	9	8
Middle Eastern/North African	1	1
Asian	0	1
Education		
Low	2	1
Medium	7	5
High	1	4
DSM Classification		
Alcohol dependence in forced remission (detention)	1	0
Personality disorder NOS	3	2
Borderline personality disorder	3	2
Depression	2	2
Adjustment disorder with mixed disturbance of emotions and conduct	1	2
Delusional Disorder or psychotic disorder NOS	0	3
PTSD	0	1
Mental Retardation	1	1
Psychosocial and environmental problems	0	1

### Data extraction

The variables selected in advance included: age, sex, ethnic group, education and DSM classification (see Table 1). Subsequently, we examined the explanation the suspect gave for the homicide act, retrospectively reported attitudes and feelings, feelings observed by forensic experts, whether the homicide was planned or not. Secondly, childhood and parenting style in the family of origin of the suspect were described. Next, personality traits, defense mechanisms, conscience, frustration-tolerance (under the heading ‘Personality’) were examined. Subsequently, psychological problems at the time of the offense or in the history of the subject were identified, as well as signs of a failure of reality testing. Finally, circumstances like substance use and stress factors (including relationship problems and divorce) and the judicial history of the suspect were described. Initially, self-description was also included in the study, but it turned out to not have been discussed in six of the twenty cases and therefore the assessment of this construct was insufficiently informative. Intelligence was also included at first, but it was not quantitatively specified how intelligence was measured. Intelligence was sometimes examined through test research and at other times estimated by the forensic expert based on the conversation with the suspect and the suspects education, which made it more difficult to compare.

### Analyses

For each case, a data capture sheet was kept regarding the reasoning on whether or not it was about revenge according to this definition. After assigning cases to ‘Revenge’ or ‘No Revenge’, the information was merged per theme in a separate document in order to give the data the same order. Issues such as childhood and youth, defense mechanisms and forensic expert-impression are usually mentioned in the reports. If this was not the case, the box in question was left empty. It was not possible to blind for Revenge versus No Revenge as this is mentioned in the case reports.

No special software program for data analysis was used, thematic analysis was performed in an iterative comparative way. Data of both groups was analyzed together and the distribution of the themes between the two groups was compared after the qualitative analysis. The thematic analysis of two independent coders was discussed in peer debriefing [21]. Two coders (Grobbink, L., Huijbregts, K.) coded the texts of the first two case-files independently, topics were then compared and consensus was reached about an initial framework. Analysis was continued by updating the coding framework after every two case-files and in discussions at research-group meetings (peer reviews). This allowed updates of the topic list in the light of emerging themes and maximized agreement concerning participants’ considerations. The themes emerged from the content and items from the files, although we made use of existing literature (see the Introduction) for further fine tuning. Existing knowledge was therefore also used when merging the codes. Also, the case reports had a certain structure so that certain aspects were always mentioned. By searching for new topics and testing predetermined topics, this method was both inductive and deductive. Data saturation was reached for both groups. Relevant themes emerging from the data were categorized in five main themes (factors in general, psychological symptoms, personality, childhood and parenting style in family of origin, and the social circumstances) and fifteen subthemes described in Table 2. Below this table, we also add the research question a main theme relates to, as formulated at the end of our introduction.

**Table 2:** Themes emerging from the data

Main themes	Subthemes
(Related research question)	
I. Factors in general (3, 4)	Causes of homicide
	Attitude and feelings towards the victim and the homicide
	Feelings observed by the forensic experts
	Planning
II. Childhood and parenting style (2)	Family of origin, childhood
III. Personality (3)	Personality traits
	Defense mechanisms
	Conscience
	Frustration-tolerance
IV. Psychological symptoms (1)	Psychological complaints
	Reality testing
V. Social circumstances (4)	Substance use
	Stress factors/reduced wellbeing
VI. Correlates (4)	Judicial history

(1) Relates to research question: Which mental health disorders were identified in the suspects?

(2) Relates to research question: What information is available regarding their personality development?

(3) Relates to research question: What is known about their personality at the time of the act?

(4) Relates to research question: Which other factors may have played a role (e.g., substance abuse or contextual factors)?

## Results

In the Results section, comparisons for the six main themes are presented between No Revenge and Revenge subjects, respectively. As mentioned above, the assessment reports were structured to always address certain topics, which are listed in Table 2. Below, we describe the results according to this structure. In a footnote (and in Table 2), we indicate the research question associated with these topics.

### Factors in general (causes, attitude and feelings towards the victim and the homicide, feelings observed by forensic experts, planning)^1^

#### Causes of homicide

In the ‘No Revenge-group’, three main reasons for the offence of the suspects were found: 1) Argument escalation, 2) Assisted suicide and 3) ‘Saving’ the other from a life that is worse than death. Escalation of an argument in a relationship in which self-defense (“I wanted him to stop”) ended fatally for the person who initiated the physical violence, assisted suicide where it is unclear whether the other person also had a death wish (but not completely implausible) and, more frequently in this sample, ‘saving’ the other from a life that is worse than death (stemming from psychosis, culturally-colored experience, such as the influence of a Djinn, the Devil or witchcraft, or a supposedly otherwise hopeless situation) as well as psychodynamic factors such as symbiosis, boundary dissolution, denial and projection (immature defense mechanisms).

In the ‘Revenge’ group, two reasons were found: 1) The feeling ‘I am not the only one who needs to feel pain’ and 2) the feeling of disrespect which cannot be tolerated (‘who does not want to listen, has to feel the consequences’). Suspects feel abandoned, humiliated, let down, and unappreciated. A partner who threatens to leave, (alleged) cheating, doubts about biological paternity and an accumulation of problems are often the immediate cause.

Table 3 shows some examples of statements made by the suspects.

**Table 3:** Examples of statements

Causes of homicide
“I had to save my daughter from the devil so that we could ascend to heaven” (Woman, 38 years old, No Revenge Group-respondent 2)
“I wanted to save him from the people who want to sexually abuse and kill him” (Woman, 42 years old, No Revenge Group-respondent 3)
“The house was cursed, my relatives are devils ” (Man, 59 years old, No Revenge Group -respondent 6)
“It’s hard to explain, but we’re a trinity, like magnets. We’re stuck together” (Woman, 41 years old, No Revenge Group -respondent 7)
“You know I can’t be alone! What are you doing to me now! She couldn’t leave me, I didn’t accept that. No one will leave me and certainly not with the children.” (Man, 42 years old, Revenge Group -respondent 9)
“If you deny me the right to be a full-fledged father, I don’t think you have the right to be a full-fledged mother either.” (Man, 38 years old, Revenge Group -respondent 5)
“She looked at other men. She’s had a really big mouth lately. I have said; enough” (Man, 68 years old, Revenge Group -respondent 6)
“I wanted him to feel the same as I felt; the same pain, fear and powerlessness” (Woman, 23 years old, Revenge Group -respondent 4)
“I warned you, now you’re dead. You wouldn’t listen. Look what you’ve done to me now, you don’t see how it’s destroying me.” (Man, 52 years old, Revenge Group -respondent 8)
Attitudes and feelings towards the accused
“I felt like I was nobody” (Man, 39 years old, Revenge Group -respondent 2)
“The better our relationship went, the better I felt about myself” (Woman, 23 years old, Revenge Group -respondent 4)

#### Attitude and feelings towards the victim and the homicide

In the ‘No Revenge-group’ suspects appear to believe that they can decide that the life of the victim is no longer worth living under the perceived circumstances. They were convinced that they were doing the right thing because of a distorted perception of reality: the victim’s fate was already terrible (for instance because of the work of the Devil), and fear and despair prospered. Children with disabilities in combination with exhaustion of the parent together with the belief that others would not be able to take care of them, can also be a non-psychotic route leading to the same consideration. Two cases appear to be outliers; during an escalated argument, one suspect had a reliving of a previous traumatic experience and in one case ‘assisted suicide’ was offered to a seriously ill partner. In the ‘No Revenge’ group, intense feelings of guilt, regret, sadness, remorse, and anger at oneself came to the fore.

In the ‘Revenge’ group, suspects experienced an accumulation of traumas and other problems. Violence was almost always experienced as a sort of settlement of an interpersonal score. Feeling abandoned is often the common denominator. Suspects were angry, offended, felt ridiculed and humiliated. They felt locked out and rejected by the world. Suspects experienced betrayal (e.g., catching the partner having extramarital sex, or doubts about biological paternity). It is noticeable that self-esteem is often linked to the relationship with a partner, in cases where the partner is murdered out of revenge. The ‘Revenge group’ frequently mentioned anger. When asked by the forensic expert, two suspects showed no remorse or guilt. One was happy that his partner was dead and that he could move on. Another said that he did not deserve a prison sentence. One person mentioned that speaking about the murder made him feel positive. Another spoke of a positive turning-point: “If it hadn’t happened then, I would still be in this shit. I am a nicer person now, I am open and dare to open my mouth now. I won’t let anyone walk all over me anymore, if my girlfriend were to tell me something now, then there’s the door.” Another said it feels good to talk about sad things from the past. Sometimes it is also not so clear how the person concerned feels about the charges, for example: ‘it seems to go past him’ (observation made by the forensic expert).

#### Feelings observed by the forensic experts

In the ‘No Revenge group’, forensic experts mentioned the following feelings: sadness, fear, upheaval, grief, and feelings of guilt that are almost unbearable and are pushed away with anger.

In the ‘Revenge group’ it was frequently noted by forensic experts that little shame and suffering were shown. Some suspects made jokes and the vibe was light-hearted. Emotions such as sadness, however, did not appear. Relatively often, suspects appeared averse to their own emotional world. Some felt wronged and felt sorry for themselves and for what happened to them.

#### Planning

Planning was denied by almost everyone.

In the ‘No- Revenge group’, there was no clear planning in seven cases. In two cases, planning is plausible because a method of committing murder/suicide was searched for on the internet. In one case, the suspect had already given signals that something was going wrong (this may indicate not so much that someone was planning, yet the offense did not come completely out of the blue).

In the ‘Revenge’ group there was no clear planning in four cases. Two suspects prepared carefully (scrupulously) and this makes planning very plausible. In one case, planning was plausible because a method was searched for on the internet. In two cases planning was denied even though there was a threat through text messages and e-mail. In one case there was a suicide note, but it was undated, making it difficult to prove planning.

### Childhood and parenting style in family of origin^2^

#### Family of origin, childhood

In both groups parenting styles were experienced as inconsistent: one authoritarian, the other accommodating and safe, or one hot-tempered and the other one calm. Little attention was paid to the individual and to the emotional needs. Physical abuse took place in both groups.

In the ‘No- Revenge group’, it is noticeable there was a limited mutual involvement in the family of origin. Parents died when the suspects were still young, or they suffered from psychological complaints as a result of which little affection and safety could be given. Systemic problems were described a number of times (e.g., extramarital relationships, a lot of fighting between parents or a child who feels that he is standing between the parents as a mediator). Several suspects struggled with attachment problems and behavioral problems at a young age, had few friends or experienced bullying at school. Being bullied at school is mentioned in half of the cases. Being bullied by a stepmother and emigration/acculturation problems were both mentioned once.

In contrast to the ‘No Revenge’ cases, being bullied by peers in school is hardly a recurring theme in the ‘Revenge’ cases. Separation of parents, early childhood trauma (including sexual and physical abuse) and alcohol use is reported. Status, norms and values ​​were important themes for various suspects involved and feelings of belittlement and humiliation are experienced. In addition, in one of the families it is described that the person concerned may have been spoiled, which may have contributed to a low frustration tolerance.

### Personality (personality traits, defense mechanisms, conscience, frustration-tolerance)^3^

#### Personality traits (identified by experts)

In the ‘No Revenge group’ a borderline personality organization [22] was identified three times. Furthermore, introversion was discussed as well as internalizing problems, sub-assertiveness and avoidant and dependent traits. There is evidence of an avoidant attachment style. Feelings of emptiness, suggestibility, fear of abandonment, an erratic emotional life with a poorly-matured identity, and mood swings were also identified. Intimacy problems and a tendency to take on the victim role are also mentioned, as well as more neurotic problems (e.g. nervousness). In addition, indications of a psychotic personality organization were found. This can partly be derived from the failing reality-testing that was reported frequently in this group, as well as schizoid traits, and a symbiotic relationship tendency (all of these can be indicator of a psychotic personality organization). In several cases, there was a psychological fusion with the other person or a symbiotic relationship. Aggression was often inhibited with a passive aggressive expression of dissatisfaction. In addition, drugs seemed to play a role sometimes, which means that failing reality-testing could in that case also be caused by drug use.

In the Revenge group, egocentrism is mentioned no less than eight times. Passive aggressive traits, dependent and avoidant traits, perfectionism, rigidity, lack of close contact, jealousy, inadequate emotion regulation, and impulsivity were also mentioned. Antisocial traits and a tendency to externalize were mentioned twice. In particular, there could be covert narcissism (such as denial of one’s own hostility and suspicion). More primary narcissism could also be deduced in one case from the description of a socially skilled façade. Narcissism manifests itself as well in materialism and search for power and prestige, egocentrism, fear of belittlement, self-centeredness, low empathy, need for recognition, difficulty with authority, low frustration tolerance, identity problems, black- and- white thinking, fantasies of grandeur and a lack of mentalization. Contact with others was instrumentally colored.

#### Defense mechanisms

Defense mechanisms that were the same in both groups: splitting, projective identification, affect isolation, externalization, idealization and devaluation, rationalization, and denial. For example, one man in the No-Revenge group denied his partner’s death (and his fault) and became angry and sad when confronted.

Furthermore, in the ‘No-Revenge group’ acting out, externalizing, rationalization, alienation, projection, reaction formation, repression, passive-aggressive complaining was present. Although there is a mixture of primitive and developed defenses, immature defense-mechanisms predominate.

In the ‘Revenge group’, defense-mechanisms as paranoid projection and vilification were seen. More primitive defenses were also identified. In one case, one could speak of a severely fluctuating level of insight and judgement.

#### Conscience (identified by experts)

In the ‘No-Revenge’ group, there was no mentioning of a disturbed function of conscience. Reasonable, intact, strict or punitive functions of conscience were described.

In the Revenge group, conscience was described as flawed (only one exception). It is insufficiently internalized, very limited, lacunar, and/or built up from outside rules commandments (e.g. faith), or it consists of a rational awareness of norms and values and fails on an emotional and behavioral level.

#### Frustration-tolerance (identified by experts)

In the ‘No- Revenge group’, the conclusion of the experts is that the level of frustration-tolerance varied; from moderate, low to strong control, and even inhibited. There seem to be an impulsive group with low frustration-tolerance and a group in which this is less evident due to aggression-inhibition. The latter group may be an extra risk because of unpredictability. Risk mainly arises when coping fails because ‘suddenly’ a completely different side of the victim emerges.

In the ‘Revenge group’, frustration-tolerance was usually rated as low. None of the subjects had an aggression breakthrough during the research itself. However, an emotional sensitivity and fragility is often observed. In one case, the frustration tolerance was estimated as being held back: ‘Frustrations built up and form the breeding ground of anger’. In some of the cases, frustrations were visible (and also more externally and verbally focused than in the No-Revenge cases), but they usually did not lead to major outbursts. The frustration-tolerance seemed to be intact somewhat more often than in the No-Revenge group.

### Psychological symptoms (psychological complaints and reality testing)^4^

#### Psychological complaints

Psychotic symptoms regularly played a role in the ‘No-Revenge’ cases. Based on delusions, the suspect has the idea that the victim’s life was no longer worth living. In particular, paranoid ideas and delusions of reference and mental disorganization were mentioned. Mood disorders were frequently mentioned in the reports (specifically in at least five of the cases). Usually it concerns sadness, feelings of powerlessness and desperation, suicidality, sleeping-problems and occasionally an agitated or excited mood. Relationship problems regularly played a role. They either led to a longer lasting pattern of overload or to an acute severe degree of distress. They were specifically mentioned in at least three of the ‘No- Revenge’ cases. Social isolation, acculturation problems, reliving of a previous traumatic experience, problems in the religious sphere, Factitious Disorder Imposed on Another (Munchausen by proxy) and substance use were all mentioned once.

In the ‘Revenge’ cases, the psychotic symptomatology was remarkably absent compared to the ‘No- Revenge’ cases. Depressive symptoms were also mentioned less often (in three out of ten cases). However, personality problems were mentioned more often (particularly the B-cluster in terms of the DSM-IV) and suspects mentioned more often having no psychological complaints (three times). Substance use and relationship problems were also mentioned more often. Remarkable is that one report mentioned that an extensive mental care history could not reverse a negative trend and another subject already showed a trend of increasing aggression before the homicide.

#### Reality testing (identified by experts)

Impaired reality testing was often the case in the ‘No- Revenge group’ (in six cases this was regarded as seriously disturbed). Three times undisturbed/intact reality testing was mentioned, one time ‘vulnerable under pressure’.

In the Revenge group, the reality testing was mostly intact; in eight cases this was undisturbed. Dissociative distortions were mentioned twice.

### Social circumstances (substance use, stress factors)^5^

#### Substance use at the time of the offense

One case involved alcohol and cannabis use and one case concerned polydrug dependence at the time of the offense in the ‘No- Revenge group’. In the Revenge group, two of the ten cases involved alcohol use at the time of the offense and one case involved a fairly large dose of oxazepam (150 mg).

#### Stress factors/reduced wellbeing at the time of the offense

The most common stress factors were similar in both groups and concerned financial problems and debts, relationship problems and the threat of divorce, work problems and loss of work, poor social position due to language problems, housing- problems or the threat of eviction, social deprivation, care for a partner or child with health- or psychological problems, somatic problems such as erectile dysfunction causing sexual problems. Often a combination of stressors seemed to be at play. Relationship problems stand out in the Revenge cases.

### Correlates (judicial history)^6^

#### Judicial history

In the ‘No- Revenge group’ one drug smuggling and two thefts are mentioned, otherwise nothing in particular. In contrast to this, there is a large degree of differentiation in the ‘Revenge’ group: five have a blank judicial history and others are familiar with insulting the police, violent crimes and attempted manslaughter, theft, drug trafficking, arson, and benefit fraud.

## Discussion

This article started with the question: Which themes play a role in the case files of people who have killed their partner and/or child out of revenge compared to people who came to this act for another reason? Applying qualitative methodology, case-files where revenge was a motive were compared to those of patients for whom this was not evident. Important differences were found between the Revenge and No-Revenge group in all characteristics, like childhood and parenting style in family of origin, personality, social circumstances, judicial history, and psychological symptoms.

Four subquestions were defined in the introduction. Below, we discuss our findings related to them.

### Subquestion 1. Which mental health disorders were identified in the suspects?

In the ‘No-Revenge’ cases, psychotic symptoms such as delusions, paranoia, and mental disorganization were prevalent, often accompanied by mood disorders, relationship problems, and social isolation. Reality testing was frequently impaired in this group.

In contrast, ‘Revenge’ cases showed fewer psychotic and depressive symptoms but more substance use. Relationship issues were often reported. Reality testing was largely intact. Personality disorders were also more prevalent in this group, as we discuss below.

### Subquestion 2. What information is available regarding the personality development of the suspects?

In both groups, parenting styles were inconsistent, with little attention to emotional needs and instances of physical abuse. In the ‘No-Revenge’ group, there was often limited family involvement due to parental loss or psychological issues, leading to attachment and behavioral problems. Bullying, particularly at school, was a common theme.

In contrast, the ‘Revenge’ group showed fewer cases of school bullying but more experiences of parental separation, early trauma, and alcohol use. Status, norms, and feelings of humiliation were prominent, and in one case, being spoiled was linked to low frustration tolerance.

The themes that emerged in the revenge group regarding personality development are in line with the existing literature, mentioned in the introduction. Earlier research [11] already pointed out the role of attachment problems, in the sense of attempting to manage a conflict of distance and proximity when a real or imaginary threat, abandonment, or rejection by the attachment figure is present. Research by Clemente and Espinosa [23], shows that infidelity is a type of conflict that is more related to indicators of revenge than to conflicts related to jealousy. Furthermore they argue that control triggers less revenge responses and elicits more dialogue, perhaps because individuals subjected to this conflict just want to end the control exerted by their partners. Liem [12] also mentioned the importance of marital problems and/or doubts about fatherhood in combination with borderline personality traits and/or a high degree of narcissism, as factors that specifically play a role in revenge in partner and/or child murders.

Oud findings regarding attachment-issues and other stressors in childhood, appear to shed some light on what predisposed the suspects in our to study to develop personality problems en other psychological problems in the here and now.

### Subquestion 3. What is known about the personality of the suspects at the time of the act?

Personality problems were frequently observed in the Revenge group, with disorders such as narcissistic personality disorder, borderline personality disorder, and early developmental issues previously linked to the need for revenge [2, 9].

Violence within this group was typically driven by the need to settle interpersonal conflicts. A fragile sense of self, coupled with immature defense mechanisms, appeared to contribute to sudden outbursts of aggression. Common personality traits associated with this group included egocentrism, perfectionism, rigidity, difficulties in maintaining close relationships, jealousy, poor emotional regulation, impulsivity, and passive-aggressive, dependent, and avoidant tendencies. Notably, not only primary, but also covert narcissism may play a significant role in these behaviors.

### Subquestion 4. Which other factors may have played a role (e.g., substance abuse or contextual factors)?

The most commonly mentioned *stress factors* were financial problems and debts, relationship problems and the threat of divorce, work problems and loss of work, poor social position due to language problems, housing-problems or the threat of eviction, social deprivation, care for a partner or child with health or psychological problems and somatic problems such as erectile dysfunction (causing sexual problems). Often a combination of stressors seemed to be at play.

### Integration of the results based on the four subquestions

Considering our previously given definition of revenge [2], the personality problems observed in the Revenge group (compared to the No-Revenge group) align with fantasies of grandeur, cognitive distortions (such as black-and-white thinking), a fragile sense of self, and immature defense mechanisms. What the results also reveal, is that after the revenge little shame and suffering was evident. Whether or not the psychological balance and sense of self was restored on the long term, we cannot answer because we were not able to speak to the suspects at a later date due to the right to privacy. Nevertheless, this could be interesting for further research.

Based on our analysis we hypothesize that in the vulnerable (primary, but also, covertly narcissistic) persons in the Revenge group, a decline in quality of life and wellbeing could remove a buffer if there is a trigger such as infidelity or an imminent breakup. Such a decline in quality of life and wellbeing (due to increasing stress factors) might lead to a ‘straw that breaks the camel’s back’ situation.

Revenge, at its core, appears to be a disruptive event that takes place during times of psychological distress and high tension, clashing with a constellation of upbringing and personality in someone with little resilience and adaptability.

This is interesting in the light of broader research on wellbeing in mental health care [24], that describes that patients can have a reasonable level of wellbeing even with a relatively high level of psychopathology. Patients with a low level of wellbeing and a high degree of psychopathology may become demoralized. Psychopathology must therefore also be viewed in the context of functioning. This does seem to be the case with the Revenge group: these persons already have (latent) personality problems and then something happens that lowers the level of wellbeing below a certain threshold, which is unbearable and can lead to revenge ideations, and sometimes to destructive acts such as homicide. Indeed, we found that numerous stressors were mentioned in the assessment reports.

Several validated questionnaires have been developed to assess functioning and wellbeing [24, 25]. We recommend studying their usefulness in treating the patients described in our paper.

### Limitations of this study

This is a small qualitative study with 20 participants so the results should be carefully interpreted. Although the suspects admitted their guilt towards the police and during the psychological investigation, the trial was yet to come. This may have influenced the openness of those involved. For example, one may fear being at a disadvantage if one confesses to planning the act. Also, medication was not coded. In retrospect, this may have been a missed opportunity.

Another possible limitation of the study concerns the difference in male-female ratio between the groups. The psychological make-up of criminal women is different from that of men [26]. However, revenge-ideations do not necessarily differ between men and women, but could also be related to the degree of narcissism. Brown [27] found that the degree of narcissism successfully distinguished between vengeful and non-vengeful people who are low in forgiveness. In people with a high degree of forgiveness, narcissism was found to be unrelated to revenge. In other words, the most revengeful people are those who have a low score for the trait forgiveness and a high score on narcissism. This was independent of gender. When we look at the two women in the Revenge group, especially the externalizing behavior (for example manipulation, anger towards others, jealousy and the need for control) stands out. Research [26] shows that women in forensic health care show more internalizing behavior problems than men, while at the same time externalizing problem behavior is also strongly present. The two women in our Revenge group, are not the ‘typical forensic women’ described in the study by Graat et al. [26], since they had few internalizing problems.

Another limitation is that we were unable to collect data on the legal outcome of the trials (for instance whether or not the suspect were deemed criminally responsible by the judge).

Finally, a strength of our approach is that we worked with two independent coders. On the other hand, a limitation of the coding is that the case files had to be divided in advance between Revenge and No Revenge, so there was no blind coding.

### Relevance to clinical practice

Our paper emphasizes the significance of revenge and revenge ideation, particularly in terms of its clinical consequences. Demoralization seems to be crucial for both the Revenge and No-Revenge groups. In patients that meet the profile of the Revenge group, clinicians should inquire about relationship problems, paranoia, and suicidality, noting that suicidality may involve aggression towards both oneself and others. Many suspects have either no history of mental health care or insufficient psychological support, possibly leading to an “illusion of mental health.” There’s may be a heightened risk of repeated behavior in the Revenge group, often linked to poor interpersonal skills and a lack of perspective-taking.

Research by Gabriel and Monaco [28] highlighted a vengeful personality characterized by a sense of perpetual need to equalize and past experiences of shame and humiliation. Homicide motivations in this group often stem from the belief that others should also experience pain.

Clinicians come across many healthy and unhealthy defense mechanisms in these patients, and it will not be easy to notice revenge ideations. Revenge motives were not always explicitly mentioned by our subjects. Maybe it was expected to be incriminating during criminal proceedings, but it may also be that ideations of revenge slumbered in the unconscious a long time.

We advise clinicians to ask about relationship issues, particularly in case of narcissistic traits and mood problems and a low level of well-being when stressors start mounting (for instance financial problems). Assessing identity development, defense mechanisms, separation problems and impulse control is essential, as ideations of revenge can serve various psychological functions, such as erasing an intolerable reality or maintaining a grandiose self-image.

The Vengeance Scale [29] could be administered to provide valuable insights in clinical settings (when using The Vengeance Scale the use of a measure of ‘social desirability’ is advised). This questionnaire could be used for an interesting follow-up study, especially when both the psychotherapist and the patient complete it, to compare whether the same estimate is made of the degree of revenge ideations. There could be more brooding on revenge than the psychotherapist suspects. In assessing the risk of recidivism, an instrument such as the Homicide Injury Scale (HIS) could be applied. This questionnaire assesses the level of ‘overkill.’ If a victim sustains more injuries, the level of overkill will be higher [30]. Previous research into overkill revealed that the length and closeness of the relationship between the victim and perpetrator increased the risk of violence escalating into murder, which was carried out with extreme and severe force [31]. This again points to the imprtance of attachment issues.

The above-mentioned theories and processes have to be taken into consideration in contacts with patients and in treatment. The main problem of revengeful patients is their inability to feel and bear their emotions and feelings. Draijer [9] recommended psychodynamic treatment in which the psychotherapist presents himself as a new “actual” object, supportive and acknowledging with regard to the original experienced injury. This therapy is typically long-term and requires the therapist to endure the patient’s complaints of constant suffering and pain. Patients may provoke the therapist to justify their own revengeful behavior [32–34]. It is a challenge for the therapist to bear the provocation, the distrust, and negative transference, and to offer an accepting environment (although with clear boundaries), and teach the patient to face his own shortcomings and losses to start the actual grieving process [9].

## Conclusion

Little theory is available on revenge and there are no widely shared diagnostic and treatment programs for vengeful patients. In the forensic field, there is a great diversity in the professional treatment of people for whom revenge plays an important role in their criminal history. Based on our analyses we would encourage clinicians to ask questions about revenge ideations in patients with a high level of (both primary and covert) narcissism wo report mood problems and declining quality of life/well-being, particularly when a possible trigger –for instance a break-up- is occurring or upcoming. There could be more brooding on revenge than the therapist suspects. Previous research suggests that if revenge ideations are identified, it is important to assess how the relationship has developed over time. If high levels of contentiousness and conflict were present, this increases the risk [35].

We hope to have made a start with theoretical findings that could be tested in further research, and which may lead to improvement of treatment plan and policy and may prevent escalations and acting out behavior.

## Data Availability

The datasets generated and/or analysed during the current study are not publicly available due to privacy-sensitive information but are available from the corresponding author on reasonable request.

## Abbreviations

NIFP: Netherlands Institute of Forensics Psychiatry and Psychology
PTSD: Post-Traumatic Stress Disorder

## Footnotes

1 Relates to the following research question: what is known about their personality at the time of the act? Which other factors may have played a role (e.g., substance abuse or contextual factors)?

2 Relates tot he following research questions: what information is available regarding their personality development?

3 Relates to research question: What is known about their personality at the time of the act?

4 Relates to research question: which mental health disorders were identified in the suspects?

5 Relates to research question: which other factors may have played a role (e.g., substance abuse or contextual factors)?

6 Relates to research question: which other factors may have played a role (e.g., substance abuse or contextual factors)?

## References

[1] Editorial staff. Vader bereidde moord op zijn zoons tot in detail voor [Father prepared murder of his sons in detail]. 2013. AD. https://www.ad.nl/binnenland/vader-bereidde-moord-op-zijn-zoons-tot-in-detail-voor~a78fe132/.

[2] Grobbink LH, Derksen J, van Marle HJ. Revenge: an analysis of its psychological underpinnings. Int J Offender Ther Comp Criminol. 2014;16:0306624X13519963. 10.1177/0306624X13519963 24441031

[3] Grobbink LH, Derksen J. Wraak [Revenge]. GZ-psychologie. 2012;4:18–23.

[4] Declercq F, Audenaert MD. A case of mass murder: personality disorder, psychopathology, and violence mode. Aggress Violent Behav. 2011;16:135–43.

[5] Declercq F, Audenaert MD. Predatory violence aiming at relief in a case of mass murder: Meloy’s criteria for applied forensic practice. Behav Sci Law. 2011;29:578–91. 21748789 10.1002/bsl.994

[6] Orth U, Montada L, Maercker A. Feelings of revenge, retaliation motive, and posttraumatic stress reactions in crime victims. J Interpers Violence. 2006;21(2):229–43. 16368763 10.1177/0886260505282286

[7] Haley WE, Strickland BR. Interpersonal betrayal and cooperation: effects on self-evaluation in depression. J Pers Soc Psychol. 1986;50(2):386–91. 3701585 10.1037//0022-3514.50.2.386

[8] Barcaccia B, Salvati M, Pallini S, et al. The bitter taste of revenge: negative affect, depression, and anxiety. Curr Psychol. 2020. 10.1007/s12144-020-00643-1.

[9] Draijer N. Moord en doodslag als pathologische wraak [Homicide as pathological revenge]. In: Goudswaard H, editor. Vergeven of vergelden; over slachtoffers van intiem geweld [Forgive or take revenge: about victims of intimate violence]. Amsterdam: Van Gennep; 2000. p. 59–80.

[10] Searles HF. The psychodynamics of vengefulness. Psychiatry. 1956;19(1):31–9. 10.1080/00332747.1956.11023029. 13310696 10.1080/00332747.1956.11023029

[11] Pistole MC. Adult attachment styles: some thoughts on closeness-distance struggles. Fam Process. 1994;33:147–59. 10.1111/j.1545-5300.1994.00147.x. 7925925 10.1111/j.1545-5300.1994.00147.x

[12] Liem M. Homicide followed by suicide: an empirical analysis (Doctoral dissertation). Utrecht: Utrecht University; 2010.

[13] Johnson C, Sachmann M. Familicide-suicide. Fam Court Rev. 2014;52:100–13. 10.1111/fcre.12067.

[14] Jackson JC, Choi VK, Gelfand MJ. Revenge: a multilevel review and synthesis. Annu Rev Psychol. 2019;70:319–45. 30609911 10.1146/annurev-psych-010418-103305

[15] Mossman D, Noffsinger SG, Ash P, Frierson RL, Gerbasi J, Hackett M, et al. American Academy of Psychiatry and the Law. AAPL practice guideline for the forensic psychiatric evaluation of competence to stand trial. J Am Acad Psychiatry Law. 2007;35(4 Suppl):S3–72. 10.1007/s12144-020-00643-1. 18083992

[16] Mumley DL, Tilbrook CE, Grisso T. Five-year research update (1996–2000): evaluations for competence to stand trial (adjudicative competence). Behav Sci Law. 2003;21:329–50. 12808694 10.1002/bsl.534

[17] Beis P, Graf M, Hachtel H. Impact of legal traditions on forensic mental health treatment worldwide. Front Psychiatry. 2022;13:876619. 10.3389/fpsyt.2022.876619. 35546946 10.3389/fpsyt.2022.876619 PMC9082492

[18] Duits N, van der Hoorn S, Wiznitzer M, Wettstein RM, de Beurs E. Quality improvement of forensic mental health evaluations and reports of youth in the Netherlands. Int J Law Psychiatry. 2012;35(5–6):440–4. 23040679 10.1016/j.ijlp.2012.09.018

[19] Van der Leij JBJ, Jackson JL, Malsch M, Nijboer JF. Residential mental health assessment within Dutch criminal cases: a discussion. Behav Sci Law. 2001;19(5–6):691–702. 10.1002/bsl.457. 11787076 10.1002/bsl.465

[20] Boon S, Yoshimura S. Revenge as social interaction: merging social psychological and interpersonal communication approaches to the study of vengeful behavior. So Pers Psychol Compass. 2020;14:e12554.

[21] Lincoln YS, Guba EG. Naturalistic inquiry. Beverly Hills: Sage Publications; 1985.

[22] Kernberg O. The development of intrapsychic structures in the light of borderline personality organization. In: Pollock G, Greenspan S, editors. The course of life: psychoanalytic contributions toward understanding personality development, vol. III. 1980. p. 349–66.

[23] Clemente M, Espinosa P. Revenge in couple relationships and their relation to the dark triad. Int J Environ Res Public Health. 2021;18(14):7653. 34300105 10.3390/ijerph18147653 PMC8304795

[24] Franken K, Lamers SM, Ten Klooster PM, Bohlmeijer ET, Westerhof GJ. Validation of the mental health continuum-short form and the dual continua model of well-being and psychopathology in an adult mental health setting. J Clin Psychol. 2018;74:1–16. 10.1002/jclp.22659 PMC6282789 29978482

[25] Ware JE, Sherbourne CD. The MOS 36-item short-form health survey (SF-36). Med Care. 1992;30(6):473–83. 1593914

[26] Graat R, Lammers S, Bloemsaat G. Sekseverschillen bij tbs-patiënten - Vrouwen gestoorder, mannen gevaarlijker? [Sex differences in TBS patients: women more disturbed, men more dangerous?]. De Psycholoog. 2011;46:10–20.

[27] Brown RP. Vengeance is mine: narcissism, vengeance, and the tendency to forgive. J Res Pers. 2004;38:576–84.

[28] Gabriel MA, Monaco GW. Getting even: clinical considerations of adaptive and maladaptive vengeance. Clin Soc Work J. 1994;22(2):165–78.

[29] Stuckless N, Goranson R. The vengeance scale: development of a measure of attitudes toward revenge. J Soc Behav Pers. 1992;7:25–42.

[30] Jordan CE, Pritchard AJ, Duckett D, Wilcox P, Corey T. Relationship and injury trends in the homicide of women across the lifespan: a research note. Homicide Stud. 2010;14:181–92.

[31] Zara G, Gino S. Intimate partner violence and its escalation into femicide: frailty thy name is ‘violence against women.’ Front Psychol. 2018;9:1777. 30319489 10.3389/fpsyg.2018.01777 PMC6168672

[32] Lane RC. The revenge motive: a developmental perspective of the life circle and treatment process. Psychoanal Rev. 1995;82:41–64. 7624453

[33] Hull JW, Lane RC, Okie J. Sexual acting out and desire for revenge. Psychoanal Rev. 1989;76:313–28. 2508141

[34] Brenman E. Cruelty and narrow-mindedness. Int J Psychoanal. 1985;66:273–81. 4044118

[35] Zara G, Freilone F, Veggi S, Biondi E, Ceccarelli D, Gino S. The medico-legal, psycho-criminological, and epidemiological reality of intimate partner and non-intimate partner femicide in North-West Italy: looking backwards to see forwards. Int J Legal Med. 2019;133:1295–307. 31016374 10.1007/s00414-019-02061-w PMC6570676

